# Regelbegehungen von Krankenhäusern durch den Öffentlichen Gesundheitsdienst: Deutschlandweite repräsentative Datenerhebung zu Struktur, Organisation und Inhalten

**DOI:** 10.1007/s00103-025-04162-x

**Published:** 2025-12-02

**Authors:** Anna Dornaika, Julia Dannenberg, Felix Droop, Steffen Engelhart, Nico T. Mutters, Alexander D. Wollkopf, PRO-OEGD study group

**Affiliations:** Institut für Hygiene und Öffentliche Gesundheit/Public Health, Universitätsklinikum Bonn, Venusberg Campus 1, 53127 Bonn, Deutschland

**Keywords:** Infektionshygienische Begehung, Standardisierung, Krankenhaushygiene, Gesundheitsamt, Infektionsprävention, Infection control inspection, Standardization, Hospital hygiene, Public health authority, Infection prevention

## Abstract

**Einleitung:**

In Deutschland werden Krankenhäuser regelmäßig und anlassunabhängig durch den Öffentlichen Gesundheitsdienst begangen, um Maßnahmen zur Prävention nosokomialer Infektionen zu überprüfen. Die Gestaltung der Regelbegehungen liegt im Ermessen der jeweiligen Gesundheitsbehörde. Ziel dieser Arbeit ist es, erstmals eine repräsentative Bestandsaufnahme von Struktur, Organisation und Inhalten der Regelbegehungen durch deutsche Gesundheitsämter (GÄ) vorzunehmen.

**Methoden:**

Im Projekt PRO-OEGD wurde im Zeitraum 12/2023–05/2024 eine Online-Befragung der deutschen GÄ durchgeführt. Die statistische Auswertung erfolgte mittels Chi-Quadrat‑, Fisher‑, Mann-Whitney- und Pearson-Korrelationstest.

**Ergebnisse:**

Die Rücklaufquote betrug 66 % (248 von 377 GÄ). Trotz föderal bedingter struktureller Unterschiede wurden viele Gemeinsamkeiten festgestellt, insbesondere hinsichtlich der Inhalte der Regelbegehungen. Strukturell sind meist ärztliche Fachkräfte, Hygienekontrolleurinnen und -kontrolleure sowie Hygienefachkräfte an den Begehungen beteiligt. Kliniken der Maximalversorgung werden häufiger als Nichtmaximalversorger mehrfach pro Jahr begangen. Meist (90 %) werden die Krankenhäuser vorab über den Termin informiert. Zwei Drittel der GÄ nutzen standardisierte Checklisten. Ein kleiner Teil (3 %) führt eine systematische Auswertung der Begehungsergebnisse mit Feedback an die Einrichtungen durch.

**Diskussion:**

Auf Grundlage der Ergebnisse wird die Entwicklung von standardisierten Checklisten angestrebt, um eine deutschlandweite Vereinheitlichung der Krankenhaus-Regelbegehungen zu ermöglichen. Dies soll zu einer effizienteren Nutzung von Personalressourcen beitragen, zukünftige Digitalisierungsinitiativen erleichtern und eine Grundlage für die Vergleichbarkeit der Ergebnisse schaffen.

**Zusatzmaterial online:**

Zusätzliche Informationen sind in der Online-Version dieses Artikels (10.1007/s00103-025-04162-x) enthalten.

## Abstract (en)

**Introduction:**

In Germany, hospitals undergo routine inspections (RIs) by public health authorities in an occasion-independent manner for infection prevention and control (IPC). Structure and organization of RIs is at the discretion of respective public health authorities (PHAs) and can therefore differ between districts. This work aims to provide the first representative overview of structure, organization, and content of RIs by German PHAs.

**Methods:**

As part of the PRO-OEGD project, representative data collection among all German PHAs was conducted between December 2023 and May 2024. Statistical analysis was performed using the chi-square, Fisher’s, Mann-Whitney-, and Pearson-correlation tests.

**Results:**

The response rate was 66% (248 of 377 PHAs). Results show many similarities despite different federal structures, for example, with regard to rating of content and hospital departments being particularly relevant for IPC. Medical professionals, hygiene inspectors, and hygiene specialists are usually involved in RIs. Maximum care providers, more often than non-maximum care providers, are inspected several times a year. Mostly (90%), hospitals are informed of the RI in advance. Two thirds of PHAs use standardized checklists. Few PHAs (3%) perform a systematic evaluation of the RI results and give feedback to the hospital.

**Discussion:**

Based on the results, a nationwide standardization of RIs using jointly agreed checklists is being pursued. Practical examples show that the use of checklists helps ensure efficient use of personnel resources before and during RIs. Standardized checklists facilitate the implementation of future digitalization initiatives while also enabling comparability of RI results.

## Einleitung

Krankenhäuser werden in Deutschland regelmäßig durch die überwachenden Gesundheitsbehörden begangen. Diese Begehungen werden auf Grundlage des Infektionsschutzgesetzes, der Hygieneverordnungen der Bundesländer sowie der Gesetze des Öffentlichen Gesundheitsdienstes (ÖGD) der Länder durchgeführt [1]. Sie dienen der Überprüfung, ob Maßnahmen zur Prävention nosokomialer Infektionen umgesetzt und so konsekutiv Infektionsrisiken minimiert werden. Hierbei ist zu unterscheiden zwischen regelmäßig wiederkehrenden, anlassunabhängigen Begehungen („Regelbegehungen“) und solchen mit konkretem Anlass, z. B. nach Meldung von hygienerelevanten Vorkommnissen. Die folgenden Ausführungen beziehen sich ausschließlich auf die anlassunabhängigen Regelbegehungen.

Die Umsetzung der infektionshygienischen Überwachung erfolgt in Deutschland meist dezentral durch die Gesundheitsämter (GÄ) der Landkreise und kreisfreien Städte. Dabei liegt die konkrete Ausgestaltung im Ermessen der einzelnen Ämter. Da bisher kein bundesweiter Standard für Regelbegehungen existiert, kann sich die Ausgestaltung von Amt zu Amt deutlich unterscheiden. Eine überregionale Vereinheitlichung der Prozesse ist bisher nicht gegeben. Das Projekt PRO-OEGD (Living *Pro*tocol für Regelbegehung durch den *ÖGD*) hat sich daher zum Ziel gesetzt, ein deutschlandweit anwendbares, standardisiertes und regelmäßig zu aktualisierendes Begehungsprotokoll für strukturierte Krankenhaus-Regelbegehungen durch den ÖGD zu erstellen.^1^ Das Projekt wird vom Bundesministerium für Gesundheit über einen Zeitraum von drei Jahren gefördert. Die Koordination erfolgt über das Institut für Hygiene und Öffentliche Gesundheit/Public Health (IHPH) des Universitätsklinikums Bonn. Weiterhin an PRO-OEGD beteiligt sind zehn Gesundheitsämter des *mre-netz regio rhein-ahr *aus Nordrhein-Westfalen und Rheinland-Pfalz: Gesundheitsamt der Stadt Bonn, der Stadt Köln, der Stadt Leverkusen, Gesundheitsamt Kreis Ahrweiler, Kreis Neuwied, Gesundheitsamt Rhein-Sieg-Kreis, Rheinisch-Bergischer Kreis, Oberbergischer Kreis, Rhein-Erft-Kreis und Kreis Euskirchen.^2^

Bei der Entwicklung von geeigneten themenbezogenen Checklisten, die das Begehungsprotokoll bilden, ist es wichtig, bereits bestehende Strukturen, Organisation und Inhalte der Begehungen zu berücksichtigen [2]. Da es bisher keine umfassenden Daten oder Publikationen hierzu gibt, wurde beschlossen, zur Statuserhebung zunächst eine deutschlandweite Umfrage unter allen Gesundheitsämtern durchzuführen. In den nachfolgenden Abschnitten werden die Methoden und Ergebnisse beschrieben und mögliche Konsequenzen diskutiert.

## Methoden

Für die Erfassung von Struktur, Organisation und Inhalten der bisherigen Krankenhaus-Regelbegehungen durch den ÖGD wurden alle deutschen GÄ angeschrieben. Der vollständige Fragebogen wurde vom PRO-OEGD-Projektteam entwickelt und durch einen modifizierten Delphi-Prozess in zwei Durchgängen unter den beteiligten Projektpartnern (IHPH und GÄ des *mre-netz regio rhein-ahr*) konsentiert.

Primär adressiert wurden Personen, die in den jeweiligen Behörden für die Krankenhausbegehungen hauptverantwortlich sind. Die GÄ wurden sowohl direkt per E‑Mail angeschrieben als auch Teilnahmeinformationen über die Landesgesundheitsbehörden, einschlägige Fachgesellschaften und Berufsverbände via E‑Mail, Newsletter, Zeitschriftenartikel und Kongressbeiträge geteilt. Die Datenerhebung fand von Dezember 2023 bis Mai 2024 statt.

Insgesamt 47 Fragen wurden in 7 Fragegruppen eingeordnet. Eine Übersicht über Fragegruppen, Fragen und Antwortmöglichkeiten befindet sich im Onlinematerial zu diesem Artikel.

Die erste Fragegruppe bestand aus Verifizierungsfragen, über die sichergestellt wurde, dass überwachungspflichtige Krankenhäuser im Einzugsgebiet des Gesundheitsamts existieren und die ausfüllende Person im jeweiligen Amt für Krankenhausbegehungen hauptverantwortlich ist.

In der zweiten Fragegruppe wurden Basisinformationen wie das jeweilige Bundesland sowie Postleitzahl, Anzahl und Größe der Krankenhäuser im Zuständigkeitsbereich erfragt. Über die Eingabe der Basisdaten des jeweiligen Amtes konnte durch die Angaben zu Bundesland und Postleitzahl ebenfalls eine Verifizierung erreicht sowie doppelt ausgefüllte Fragebögen verhindert werden.

Inhalt der dritten Fragegruppe „Personalstruktur“ waren Fragen zur Teamstruktur sowie optional Schätzungen zur Anzahl der Personen, die von Amtsseite aus mit Regelbegehungen befasst waren. Ebenfalls optional konnte der geschätzte jährliche Zeitaufwand in Vollzeitarbeitstagen bezogen jeweils auf ärztliches und nichtärztliches Personal angegeben werden.

Die vierte Fragegruppe „Ablauf und organisatorische Aspekte“ beinhaltete Fragen zur Begehungsfrequenz (die Phase der COVID-19-Pandemie explizit ausgenommen), zur Begehungsstruktur, zum beteiligten Personenkreis, zu Vor- und Nachbereitung der Begehungen sowie zur Dokumentation der Ergebnisse.

In der fünften Fragegruppe „Inhalt von Regelbegehungen“ sollten insbesondere verschiedene Aspekte des Infektionsschutzes sowie Abteilungen bzw. Bereiche des Krankenhauses hinsichtlich ihrer Relevanz für Krankenhaus-Regelbegehungen beurteilt werden. Diese wurden hierfür anhand einer 5‑stufigen Likert-Skala (5 = sehr wichtig, 4 = wichtig, 3 = indifferent, 2 = eher unwichtig, 1 = sehr unwichtig) bewertet. Bei den Abteilungen der Krankenhäuser gab es zusätzlich die Möglichkeit anzugeben, dass diese nicht im eigenen Zuständigkeitsbereich vorhanden ist. Diese Antwortmöglichkeit wurde in der Auswertung mit 0 Punkten bewertet. Für die Auswertung wurde jeweils das arithmetische Mittel gebildet.

Der Fragebogen schloss mit Fragegruppe sechs und sieben, die aus Zusatzfragen zu Netzwerken gegen multiresistente Erreger und einem freien Kommentarfeld für Ergänzungen bestand. Diese beiden Fragegruppen waren ebenfalls optional auszufüllen. Die Auswertung dieser Fragegruppen wird hier nicht weiter behandelt.

Die Umfrage wurde online über Lime Survey (Lime Survey Cloud©, Version 6.14.3) durchgeführt. Datenbearbeitung und Auswertung erfolgten über Microsoft Excel 2016 (Microsoft Corporation ©) sowie R (Version 4.3.3) und RStudio (Version 2025.05.0). Die statistische Auswertung erfolgte mithilfe des Chi-Quadrat-Tests, des Fisher-Tests, des Mann-Whitney-Tests und des Korrelationstests nach Pearson (Signifikanzniveau jeweils 5 %).

## Ergebnisse

Insgesamt wurden 279 Fragebögen vollständig ausgefüllt. Nach Bereinigung konnten 248 Fragebögen in die Auswertung eingeschlossen werden. Bei 377 Gesundheitsämtern in Deutschland entspricht dies einer Rücklaufquote von ca. 66 % [3]. Die Umfrage berücksichtigte wesentliche Aspekte der Krankenhaus-Regelbegehungen, die im Folgenden dargestellt sind.

### Basisdaten der teilnehmenden Gesundheitsämter

**Abb. 1:**
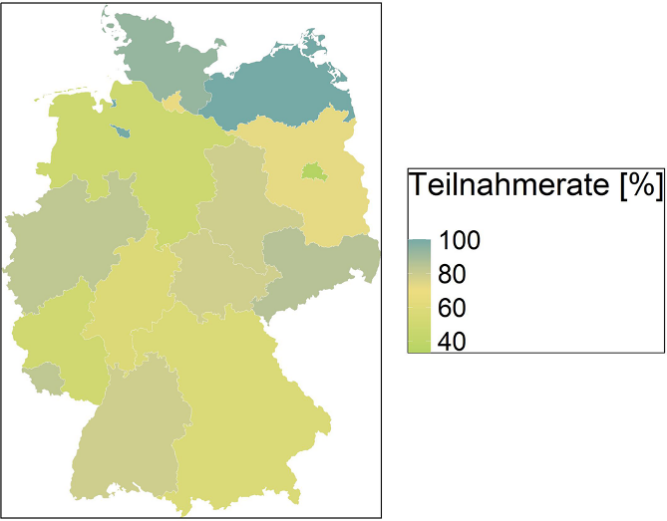
Teilnahmerate der Gesundheitsämter aufgeteilt nach Bundesländern

An der Datenerhebung nahmen GÄ aus allen Bundesländern teil (Teilnahmerate nach Bundesland: Abb. 1). Insgesamt sind die GÄ, die ihre Daten zur Verfügung gestellt haben, für die Überwachung von 1392 Krankenhäusern zuständig, darunter 85 Universitätskliniken und Maximalversorger, 143 Krankenhäuser mit ≥ 500 Betten, 344 Krankenhäuser mit 250-499 Betten und 820 Krankenhäuser mit < 250 Betten.

### Personalstruktur bei Regelbegehungen

68 % der teilnehmenden GÄ gaben an, dass bei ihnen gemeinsame Teams für Begehungen sowohl von Krankenhäusern als auch von anderen zu überwachenden Einrichtungen, z. B. Pflegeeinrichtungen, bestehen. 26 % der Ämter bilden spezialisierte Teams für die Regelbegehungen unterschiedlicher Gesundheitseinrichtungen. Vereinzelte GÄ gaben an, dass keine festen Teamstrukturen für Regelbegehungen etabliert sind und diese teilweise von Einzelpersonen umgesetzt werden.

**Abb. 2:**
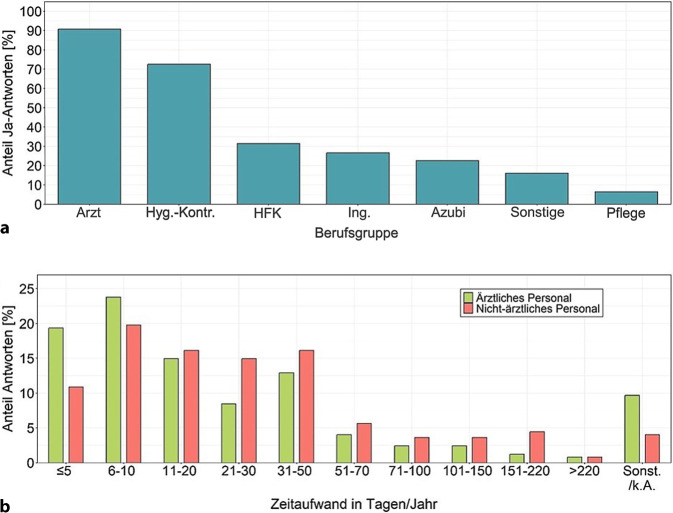
Berufsgruppen und Zeitaufwand bei Krankenhaus-Regelbegehungen. **a** Beteiligte Berufsgruppen im Gesundheitsamt (Antworten: *n* = 248). **b** Geschätzter Zeitaufwand für ärztliches und nichtärztliches Personal (Antworten: *n* = 237 bzw. 247). Erklärungen: *Arzt* Arzt/Ärztin; *Hyg.-Kontr.* Hygienekontrolleur/-kontrolleurin; *Azubi* Auszubildende; *Ing.* Ingenieur/Ingenieurin; *HFK* Hygienefachkraft; *Sonst.* Sonstige; *k.* *A.* keine Angabe

Amtsseitig sind verschiedene Berufsgruppen an Regelbegehungen beteiligt (Abb. 2a). Ärztinnen und Ärzte, Hygienekontrolleurinnen und -kontrolleure sowie Hygienefachkräfte (HFK) waren hierbei am häufigsten vertreten. Ebenfalls wurden (Hygiene‑)Ingenieurinnen und Ingenieure, Auszubildende und Mitarbeitende in der Pflege genannt. Weitere beteiligte Berufsgruppen wurden unter Sonstiges zusammengefasst, z. B. Wissenschaftlerinnen und Wissenschaftler aus den Bereichen (Mikro‑)Biologie, Biochemie und Gesundheitswissenschaften, medizinische Fachangestellte, Verwaltungsangestellte sowie Apothekerinnen und Apotheker.

Unterteilt nach ärztlichem und nichtärztlichem Personal wurde der Zeitaufwand für Krankenhaus-Regelbegehungen in Vollzeitarbeitstagen pro Jahr geschätzt. Abb. 2b gibt einen Überblick über die Antworten, wobei ein Großteil der GÄ (83 %, *n* = 197) den Zeitaufwand des ärztlichen Personals mit Vorbereitung, Durchführung und Nachbereitung der Krankenhaus-Regelbegehungen auf bis zu 50 Vollzeitarbeitstage pro Jahr schätzt. Auch beim nichtärztlichen Personal wird der Zeitaufwand in 78 % der Fälle (*n* = 193) auf bis zu 50 Vollzeitarbeitstage pro Jahr geschätzt. Ein geringer Teil der GÄ (5 %, *n* = 11) geht für ärztliches Personal von > 100 Vollzeitarbeitstagen pro Jahr aus, während es bei nichtärztlichem Personal fast doppelt so viele sind (9 %, *n* = 22). Der Zeitaufwand sowohl für ärztliches als auch nichtärztliches Personal korreliert mit der Gesamtzahl der Krankenhäuser im Zuständigkeitsbereich der GÄ (Korrelation 0,5; *p* < 0,001 und 0,37; *p* < 0,001). Dieser Zusammenhang besteht ebenfalls, wenn ausschließlich die Anzahl der Universitätskliniken/Maximalversorger betrachtet wird (0,39; *p* < 0,001 und 0,28; *p* < 0,001).

Berufsgruppenübergreifend wurde von den teilnehmenden GÄ geschätzt, wie viel Personal in Vollzeitäquivalenzstellen (VZÄ) pro Jahr für Krankenhausregelbegehungen benötigt werden. Die meisten GÄ schätzen den Personalbedarf auf 2 bis < 3 VZÄ (36 %, *n* = 90) und 3 bis < 4 VZÄ (22 %, *n* = 54). In 16 % der GÄ (*n* = 40) wird der Personalbedarf auf < 2 VZÄ geschätzt, während 22 % (*n* = 54) den Bedarf auf 4 bis < 9 VZÄ schätzen. Ein geringer Teil der GÄ (1 %, *n* = 3) sieht den Personalbedarf bei ≥ 9 VZÄ.

### Struktur und Organisation von Regelbegehungen

**Abb. 3:**
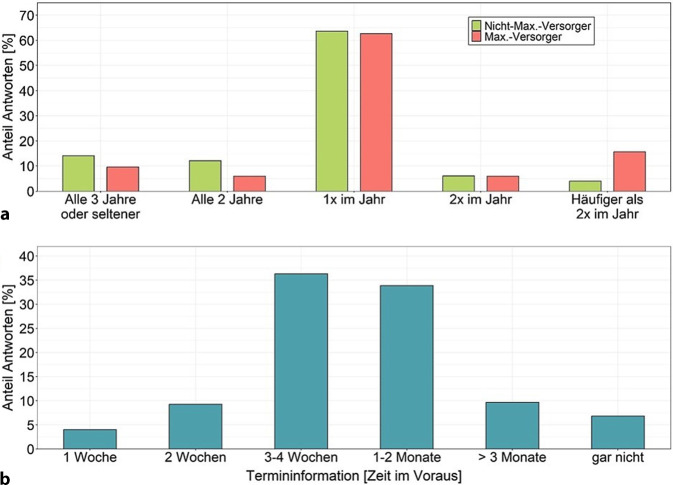
Häufigkeit der Krankenhaus-Regelbegehungen und Zeitpunkt der Termininformation. **a** Begehungsintervalle in Abhängigkeit von der Versorgungsstufe. Signifikanter Unterschied zwischen Maximalversorgern und Nichtmaximalversorgern (*p*-Wert Chi-Quadrat-Test: 0,004*). Maximalversorger *n* = 83, Nichtmaximalversorger *n* = 248. **b** Vorabinformation über den Begehungstermin. *n* = 248

Der Großteil der GÄ begeht die Einrichtungen jährlich (bei Max.-Versorger 63 %, *n* = 52; bei Nicht-Max.-Versorger 64 %, *n* = 159; Abb. 3a). Allerdings werden Maximalversorger eher häufiger als einmal im Jahr und Nichtmaximalversorger seltener als einmal im Jahr begangen (*p* = 0,004).

90 % aller GÄ teilen den jeweiligen Krankenhäusern das genaue Datum der Regelbegehung vorab mit. 3 % geben an, den Termin grob oder nur die Kalenderwoche im Vorfeld zu kommunizieren. 7 % geben keine Termine vor Regelbegehungen bekannt. Abb. 3b stellt dar, wie weit im Voraus den Krankenhäusern der Begehungstermin mitgeteilt wird. In ca. 80 % der Fälle sind dies 3 Wochen oder mehr, wobei mit 36 % am häufigsten 3–4 Wochen angegeben wurde.

Das Thema der Regelbegehung wird den Krankenhäusern von 49 % der GÄ im Vorfeld mitgeteilt. 67 % der GÄ fordern im Vorfeld der Regelbegehung Unterlagen von den Einrichtungen an, beispielsweise Hygienepläne, Surveillance-Daten, Antibiotic-Stewardship-(ABS-)Daten, Protokolle der Hygienekommissionssitzungen und Selbstauskünfte (Antworten: *n* = 166). Mehr als ein Drittel (36 %, *n* = 89) der GÄ führt vor der Regelbegehung ein separates Vorgespräch mit der zu begehenden Einrichtung.

Hinsichtlich der Begehungsstruktur bei Nichtmaximalversorgern verfolgen 56 % der GÄ (*n* = 139) und bei Maximalversorgern 55 % der GÄ (*n* = 46) das Konzept von Schwerpunktbegehungen. Hierbei erfolgen alle Begehungen der Krankenhäuser mit einem Themenschwerpunkt, welcher mit größerer Detailtiefe überprüft wird. Diesbezüglich gibt es keinen signifikanten Unterschied bei der Begehung von Universitätskliniken/Maximalversorgern und anderen Krankenhäusern (*p* = 0,899). Des Weiteren werden von den meisten GÄ Bereiche begangen, in denen zuvor Probleme in Bezug auf Infektionsprävention aufgefallen sind (84 % bei Max.-Versorgern, *n* = 70; 89 % bei Nicht-Max.-Versorgern, *n* = 221) oder neue bzw. sanierte Bereiche des Krankenhauses (80 % bei Max.-Versorgern, *n* = 66; 81 % Nicht-Max.-Versorgern, *n* = 201).

Bei der Begehung von Maximalversorgern gaben die GÄ in 47 % der Fälle (*n* = 39) an, jede Teilklinik bzw. jedes Institut einzeln zu begehen. In 7 % (*n* = 6) der Fälle werden jeweils einzelne Zentren begangen, die mehrere Kliniken zusammenfassen, z. B. Kopfzentrum oder Eltern-Kind-Zentrum, und wiederum 19 % (*n* = 16) begehen ein Universitätsklinikum bzw. einen Maximalversorger als Ganzes. 23 % (*n* = 19) gaben über eine Freitextangabe andere Modelle für die Begehungen von Maximalversorgern an, wobei am häufigsten Begehungen mit Bezug auf einen thematischen Schwerpunkt (89 %, *n* = 17) genannt wurden.

Im Vorfeld von Begehungen erfolgt der Dokumentenaustausch zwischen Amt und Krankenhaus auf unterschiedlichen Wegen. Am häufigsten werden dabei E‑Mails genutzt (71 %, *n* = 118), gefolgt vom analogen Postweg (33 %, *n* = 55), Cloud-basierten Systemen (28 %, *n* = 46) und zu einem geringen Teil dem Fax (7 %, *n* = 11).

Bezüglich der Dokumentation teilen 95 % der teilnehmenden GÄ mit, die während der Begehung erhobenen Beobachtungen auf Papier zu dokumentieren. 78 % führten zusätzlich Fotodokumentationen durch. Mobile Endgeräte wurden bisher eher wenig genutzt (15 %), allerdings wurde in einigen Fällen erklärt, sich im Beschaffungsprozess für mobile Endgeräte zu diesem Zweck zu befinden. Bei digitaler Dokumentation gaben zwei Drittel der Befragten an, Office-Anwendungen zu nutzen, während 22 % PDF-Dateien verwendeten. Vereinzelt berichteten Ämter, kommunale oder Gesundheitsamt-bezogene Software zur Dokumentation anzuwenden (*n* = 2).

Ungefähr zwei Drittel (64 %, *n* = 158) der teilnehmenden GÄ erklärten, während Begehungen standardisierte Checklisten zu nutzen. In 63 % der Fälle (*n* = 99) wurden diese Checklisten intern im Gesundheitsamt erstellt, während in 30 % diese durch das jeweilige Landesgesundheitsamt vorgegeben wurden. In 4 % der Fälle werden Checklisten anderer GÄ und anderer Behörden für Begehungen genutzt.

Alle befragten GÄ erklärten, im Anschluss an eine Begehung einen Bericht zu erstellen. 88 % der Ämter geben den Bericht vollständig an das begangene Krankenhaus weiter. Die GÄ, die den Bericht nicht vollständig weitergeben, gaben an, der Einrichtung zumindest einen Teil des Berichts, der die notwendigen auszuführenden Maßnahmen enthält, zur Verfügung zu stellen.

**Abb. 4:**
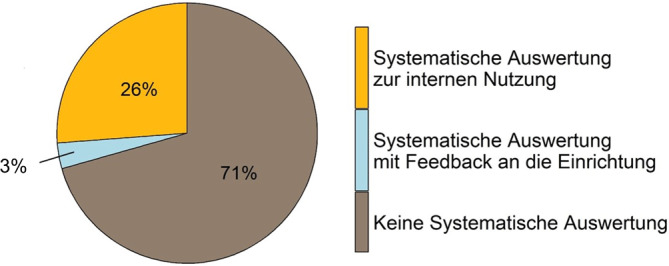
Systematische Auswertung von Regelbegehungen. *n* = 248

Rund 29 % (*n* = 73) der GÄ werten die Begehungsergebnisse systematisch aus. Der Großteil davon (26 %, *n* = 65) nutzt die Ergebnisse lediglich intern, während 3 % (*n* = 8) die Ergebnisse der Auswertung an die Einrichtungen zurückspiegeln (Abb. 4).

Sofern eine systematische Auswertung erfolgt, wurde von einigen GÄ angegeben, diese anhand selbst erstellter Checklisten, Themenschwerpunkte der Begehungen oder Strukturdaten zu Surveillance- und ABS-Themen vorzunehmen.

**Abb. 5:**
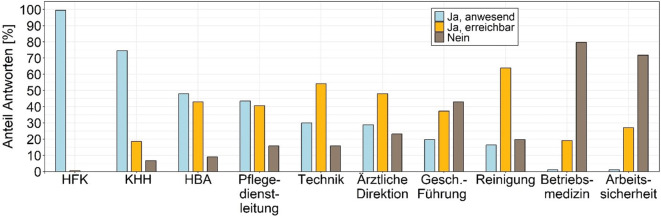
Berufsgruppen, die nach Vorgaben der Gesundheitsämter bei Regelbegehungen anwesend sein müssen (Antworten: *n* = 177). *HBA* hygienebeauftragte Ärztinnen und Ärzte; *HFK* Hygienefachkraft; *KHH* Krankenhaushygienikerinnen/-hygieniker; *Gesch.-Führung* Geschäftsführung

Mehr als zwei Drittel aller teilnehmenden GÄ geben vor, welches Personal seitens der Krankenhäuser bei einer Regelbegehung anwesend bzw. erreichbar sein sollte (Abb. 5). Hierbei wurde am häufigsten die Anwesenheit von HFK, Krankenhaushygienikerinnen und -hygieniker sowie der hygienebeauftragten Ärztinnen und Ärzte gefordert. Hygienebeauftragte Pflegekräfte standen nicht zur Vorauswahl, da sie nur stationsbezogen tätig sind, wurden aber in einem Fall ergänzend genannt. Arbeitssicherheit und Betriebsmedizin müssen laut Auskunft der meisten GÄ nicht an der Regelbegehung beteiligt sein.

### Inhalte von Regelbegehungen

Die teilnehmenden GÄ wurden gebeten, ihre Einschätzung zur Relevanz verschiedener Themen im Kontext von Regelbegehungen zu geben. Von den 36 vorgegebenen Themen wurden im Mittel am höchsten bewertet:

- Isolierungsmaßnahmen (Bewertung im Mittel: 4,8 von 5,0),
- eine hinreichende Ausstattung mit persönlicher Schutzausrüstung beim Umgang mit multiresistenten Erregern (4,8), die Kontrolle der Umsetzung von angeordneten Maßnahmen aus dem Vorjahr (4,8) sowie
- die Lagerung von Medizinprodukten (4,7).

Am wenigsten wichtig im Rahmen von Krankenhaus-Regelbegehungen erachtet wurden:

- Stellen- und Personalschlüssel (3,6),
- Pläne für die Notversorgung (z. B. Strom, Wasser; 3,6) sowie
- Aspekte des Hitzeschutzes (3,2).

Ergänzend zu den 36 vorgegebenen Aspekten wurden im Freitext-Kommentarfeld die Prüfung von Schulungs- und Belehrungsnachweisen des Personals (*n* = 12), die Haltbarkeit von Medikamenten und Medizinprodukten sowie deren Aufbereitung (*n* = 22) und Prozessbeobachtungen (*n* = 16) genannt.

**Abb. 6:**
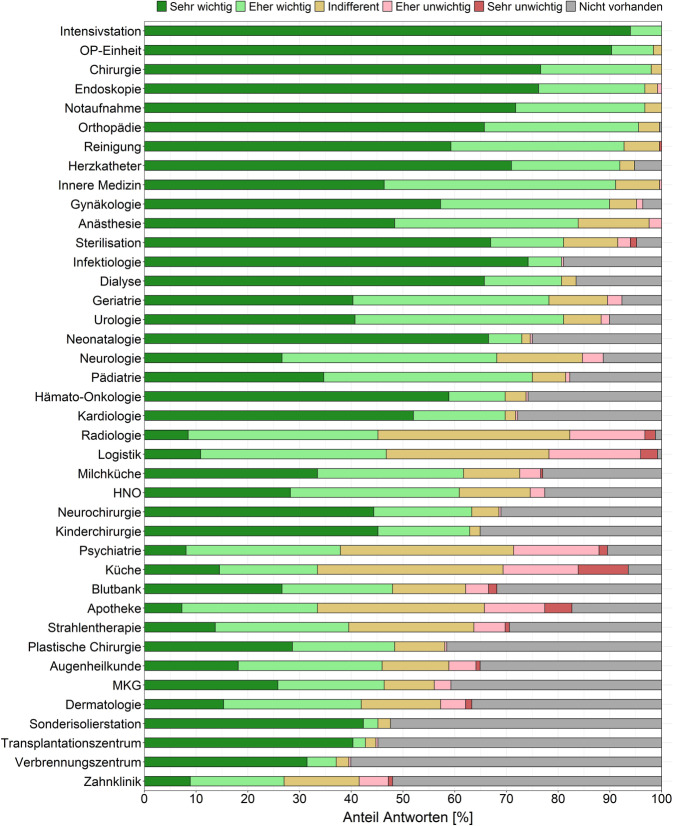
Bewertung von Abteilungen bzw. Bereichen in Bezug auf ihre Relevanz bei Krankenhaus-Regelbegehungen. Absteigend sortiert nach Mittelwert der Bewertung. (*n* = 248). *HNO* Hals-Nasen-Ohren-Heilkunde; *MKG* Mund-Kiefer-Gesichts-Chirurgie

Nach gleichem Bewertungsprinzip wurden die GÄ gebeten, Abteilungen und Bereiche einer Klinik entsprechend ihrer Relevanz bei einer Regelbegehung zu bewerten (Abb. 6). Als am wichtigsten wurden dabei Intensivstationen (Mittelwert: 4,9), OP-Bereiche (4,9), Chirurgie (4,7) sowie Endoskopie-Abteilungen (4,7) angesehen. Die niedrigsten Mittelwerte hatten Transplantationszentren (2,2), Verbrennungszentren (1,9) und Zahnkliniken (1,7).

## Diskussion

### Durchführung von Krankenhaus-Regelbegehungen durch Gesundheitsämter in Deutschland

Die vorliegende Studie untersucht nach unserer Kenntnis erstmalig systematisch bundesweit Struktur, Organisation und Inhalte von Krankenhaus-Regelbegehungen durch den ÖGD. Mit einer Rücklaufquote von insgesamt 66 % und Beteiligung von Ämtern aus allen 16 Bundesländern konnte eine repräsentative Anzahl an GÄ befragt werden.

Die Statuserhebung zeigt sowohl bei der Struktur als auch hinsichtlich der Organisation und Bewertung inhaltlicher Aspekte viele Gemeinsamkeiten zwischen den GÄ. Über 60 % der GÄ begehen die Krankenhäuser jährlich, unabhängig von deren Größe oder Versorgungsstufe. In den Gesetzen über den Öffentlichen Gesundheitsdienst der Länder, per Erlass durch Landesbehörden oder durch andere Empfehlungen ist zwar festgelegt, dass Regelbegehungen von Krankenhäusern „regelmäßig“ erfolgen sollen; eine Vorgabe, dass dies jährlich zu erfolgen hat, ist aber nur vereinzelt vorhanden, beispielsweise in einem Erlass des Ministeriums für Arbeit, Gesundheit und Soziales in Nordrhein-Westfalen (Aktenzeichen VB4-2022-0016341). Die im Rahmen dieser Studie erhobenen Daten zeigen jedoch auch signifikante Unterschiede zwischen den Begehungsfrequenzen von Maximalversorgern, die meist mehrmals im Jahr, und Nichtmaximalversorgern, die häufiger nur alle zwei Jahre oder seltener begangen werden. Mutmaßlich lässt sich dies durch eine risikobasierte Abwägung der GÄ aufgrund des unterschiedlichen Risikoprofils der Einrichtungen begründen [4]. Vor dem Hintergrund knapper Personalressourcen könnte dies auch als Zeichen einer konsekutiven Priorisierung gedeutet werden [5].

Bezogen auf das Terminmanagement für Regelbegehungen scheinen sich in der Praxis angekündigte Regelbegehungen bewährt und durchgesetzt zu haben [6]. So kann sichergestellt werden, dass die für die Begehung relevanten Personen anwesend und vorbereitet sind, was eine effektive Ressourcennutzung auf beiden Seiten ermöglicht.

Etwas mehr als die Hälfte der GÄ setzt bei Begehungen einen bestimmten inhaltlichen Schwerpunkt. So können jährlich wechselnde Themen abdeckt und gezielt entsprechende infektionspräventive Maßnahmen des jeweiligen Krankenhauses vertieft analysiert werden.

Um Begehungen möglichst reibungslos im laufenden Betrieb durchzuführen, ist ein kleiner und ausgewählter Teilnehmerkreis sinnvoll. Über die zu beteiligenden Personen herrscht bei den GÄ relativ hohe Übereinstimmung.

In der Bewertung der inhaltlichen Aspekte wurden nahezu alle vorgegebenen Themen von über 70 % der GÄ als eher wichtig bis sehr wichtig eingeschätzt. Einzig das Thema Hitzeschutz wurde im Kontext von Infektionsschutz-Begehungen seltener als relevant bewertet. Der hohe Anteil der als wichtig eingestuften Punkte könnte einerseits in der Vorauswahl der Themen begründet sein, andererseits aber auch den Wunsch ausdrücken, eine möglichst umfassende Erfassung und damit detaillierte Bewertung der Einrichtungen durchführen zu wollen.

Neben der Überprüfung von Struktur- und Ergebnisparametern stellen auch Prozessbeobachtungen im Rahmen von Regelbegehungen einen wichtigen Aspekt dar, da diese die praxisnahe Überprüfung umgesetzter Hygienemaßnahmen erlauben und Prozesse einen erheblichen Einfluss auf das Risiko nosokomialer Infektionen haben können. Im Rahmen der Begehung seitens der GÄ ist dabei von Vorteil, dass durch die externe Auditierung eine möglicherweise neutralere Beurteilung des aktuellen Zustandes erfolgt. Eine Untersuchung, bei der Mitarbeitende untereinander Messungen der Händehygiene-Compliance durchführten, zeigte, dass sie bei ihrer eigenen Berufsgruppe signifikant höhere Compliance-Raten ermittelten als bei Mitarbeitenden anderer Berufsgruppen [7]. Die Autorinnen und Autoren führen dies auf eine positivere Bewertung innerhalb der eigenen Berufsgruppe zurück.

Die Umsetzung von standardisierten Prozessbeobachtungen im Rahmen der Krankenhaus-Regelbegehung ist je nach Stichprobengröße mit einem hohen Zeit- und Personalaufwand verbunden. Zudem ist eine entsprechende Qualifikation des prüfenden Personals zu gewährleisten, um fachliche Aspekte hinreichend berücksichtigen und einordnen zu können sowie eine standardisierte Bewertung der Prozesse zu ermöglichen. Dies schließt neben der Kenntnis praktischer Handlungsabläufe auch Erfahrung in der infektionshygienischen Überwachung ein.

Eine Prozessbeobachtung kann für Krankenhausmitarbeitende eine ungewohnte Situation darstellen, in der sich die Durchführung des Prozesses in negativer (z. B. aufgrund von Stress) oder gegebenenfalls auch positiver Weise (z. B. durch höheres Aufmerksamkeitslevel) von der gewohnten Art unterscheidet. Dieser bekannte Einfluss, auch als „Hawthorne-Effekt“ bezeichnet, führt beispielsweise im Bereich der Händehygiene zu tendenziell höheren Compliance-Raten, wobei jedoch nicht davon ausgegangen werden kann, dass die Wirkung über den Beobachtungszeitraum hinausgeht [8]. Somit bleibt unklar, ob Prozessbeobachtungen allein einen relevanten Effekt auf den Hygienealltag im Krankenhaus haben. In Kombination mit der Bewertung von Surveillance-Daten ist jedoch vorstellbar, Struktur‑, Prozess- und Ergebnisparameter in eine umfassende Infektionsschutzüberwachung mit einzubeziehen.

### Die Nutzung von Checklisten und deren Standardisierung

Die Erhebung zeigt, dass zwei Drittel der GÄ standardisierte Checklisten nutzen, wobei die Mehrzahl davon im eigenen Amt erstellt wurde. Grundsätzlich sind Checklisten bei Begehungen hilfreich, da besonders wichtige Aspekte aktiv berücksichtigt werden können und eine standardisierte Erfassung möglich ist.

Im Rahmen der Infektionsprävention in Gesundheitseinrichtungen spielen Checklisten auch für interne Hygiene-Audits eine Rolle, was sich an internationalen Beispielen illustrieren lässt. Der National Health Service (NHS) als staatliches Gesundheitssystem im Vereinigten Königreich stellt Checklisten für interne Hygiene-Audits zur Verfügung und empfiehlt unter anderem den Betreibern von Gesundheitseinrichtungen die Überprüfung der Hygienestandards in Händehygiene und anderen Themenbereichen in regelmäßigen Abständen [9]. Die Centers for Disease Control and Prevention (CDC) in den USA haben in Zusammenarbeit mit der Association for Professionals in Infection Control and Epidemiology, Inc. (APIIC) ebenfalls Checklisten zu thematischen Modulen wie Harnwegskatheter-assoziierten Infektionen erstellt, die in den Einrichtungen zur Infektionsprävention genutzt werden können [10]. Zu dem Schluss, dass Checklisten im Themenfeld der Infektionsprävention zu einer Verbesserung der Hygienestandards führen, kommt auch eine kanadische Studie, in der konsentierte Checklisten für interne Begehungen genutzt wurden [11].

Die Erstellung solcher Checklisten ebenso wie das notwendige Nachhalten von Aktualisierungen erfordern Zeit und umfassende Kenntnisse auf dem Gebiet der Hygiene und des Infektionsschutzes. Durch Bündelung in zentral aktualisierte und durch Expertinnen und Experten konsentierte Checklisten könnten hier wertvolle Ressourcen in den GÄ geschont werden. Zudem besteht die Möglichkeit, dabei besonders wichtige und prüfenswerte Indikatoren hervorzuheben. Eine vollständige Erfassung aller Faktoren mit Einfluss auf die Entstehung nosokomialer Infektionen erscheint in der Praxis wenig realistisch.

Zusätzlich könnte eine Standardisierung der Krankenhaus-Regelbegehungen den laufenden Digitalisierungsprozess unterstützen und den Anteil der papierbasierten Dokumentation von derzeit über 90 % senken. Aktuell wird deutschlandweit eine Vielzahl an Projekten durchgeführt, u. a. mit dem Ziel, Krankenhausbegehungen zu digitalisieren. Einige Projekte dazu sind auf dem Portal „Digitales Gesundheitsamt“ des Bundesministeriums für Gesundheit zu finden.^3^

Neben der Schonung von Ressourcen in den GÄ und der Möglichkeit zur einfacheren Digitalisierung bietet eine standardisierte Datenerhebung auch verbesserte Möglichkeiten zur systematischen Auswertung auf lokaler oder überregionaler Ebene, was derzeit nur von wenigen Ämtern genutzt wird. Perspektivisch könnte hiervon auch die Gesundheitsberichterstattung profitieren.

## Fazit

Die vorliegende repräsentative Statuserhebung liefert erstmals einen deutschlandweiten Einblick in Struktur, Organisation und Bewertung der Inhalte von Krankenhaus-Regelbegehungen durch GÄ in Deutschland. Die Ergebnisse zeigen, dass überregional zahlreiche Gemeinsamkeiten existieren, jedoch nur wenige GÄ die Begehungsergebnisse systematisch auswerten. Auf dieser Grundlage werden im weiteren Verlauf des Projektes PRO-OEGD konsentierte Checklisten entwickelt und evaluiert. Dies bietet die Möglichkeit, wertvolle Ressourcen in den GÄ zu schonen und eine Digitalisierung der Begehungsprozesse zu erleichtern.

## Supplementary Information

Fragenbogen der PRO-OEGD Umfrage unter allen Gesundheitsämtern zu Regelbegehungen von Krankenhäusern in Deutschland

## Data Availability

Die während der vorliegenden Studie erzeugten und/oder analysierten Datensätze sind aus Gründen des Datenschutzes nicht öffentlich zugänglich, können aber auf begründete Anfrage von den entsprechenden Autorinnen und Autoren angefordert werden.

## Footnotes

1 https://www.mre-rhein-ahr.net/projekte/pro-oegd/. Zugegriffen: 02.09.2025.

2 https://www.mre-rhein-ahr.net/. Zugegriffen: 02.09.2025.

3 https://gesundheitsamt-2025.de/. Zugegriffen: 02.09.2025.
