# Supplementary material for: Regular inspections of hospitals by the public health service: nationwide representative data collection on structure, organization, and content in Germany
Source: Bundesgesundheitsblatt Gesundheitsforschung Gesundheitsschutz. 2025 Dec 2;69(1):43–50. [Article in German] doi: 10.1007/s00103-025-04162-x (PMC12764564; doi:10.1007/s00103-025-04162-x)
Supplement: Supplementary file 1 — Fragenbogen der PRO-OEGD Umfrage unter allen Gesundheitsämtern zu Regelbegehungen von Krankenhäusern in Deutschland [file 103_2025_4162_MOESM1_ESM.docx]

**Onlinematerial**

Fragenbogen der PRO-OEGD Umfrage unter allen Gesundheitsämtern zu Regelbegehungen von Krankenhäusern in Deutschland

| PRO-OEGD Umfrage aller Gesundheitsämter zu Regelbegehungen von Krankenhäusern in Deutschland | | |
| --- | --- | --- |
| Frage-gruppe | Frage | Antworten |
| **A** | **Allgemeines** | |
| A | Befinden sich in Ihrem Kreis/Ihrer Stadt Krankenhäuser, die Ihrer Überwachung unterliegen und die Sie (bzw. Ihr Amt) regelmäßig begehen | *Einfachauswahl*   - Ja - Nein |
| A | Sind Sie für die Begehung von Krankenhäusern verantwortlich? | *Einfachauswahl*   - Ja - Nein |
| A | Wenn nein: Wurde Ihnen das Ausfüllen der Umfrage delegiert? | *Einfachauswahl*   - Ja - Nein |
| **B** | **Basisdaten Gesundheitsamt** | |
| B | In welchem Bundesland befindet sich Ihr Gesundheitsamt? | *Einfachauswahl*  Baden-Württemberg; Bayern; Berlin; Brandenburg; Bremen; Hamburg; Hessen; Mecklenburg-Vorpommern; Niedersachsen; Nordrhein-Westfalen; Rheinland-Pfalz; Saarland; Sachsen-Anhalt; Sachsen; Schleswig-Holstein; Thüringen |
| B | Wie lautet die Postleitzahl Ihres Gesundheitsamts (Hauptanschrift)? | *numerische Angabe* |
| B | Wie viele Krankenhäuser liegen in Ihrem Zuständigkeitsbereich? *Bitte beachten Sie: Wenn sich in Ihrem Kreis/Ihrer Stadt Krankenhäuser mit mehreren Betriebsstätten befinden, zählt jeder Standort einzeln. Tragen Sie bitte eine 0 ein, wenn sich kein Krankenhaus der entsprechenden Kategorie in Ihrer Zuständigkeit befindet.* | *Numerische Angabe, jeweils für:*   - Universitätskliniken/Maximalversorger - Kliniken mit ≥ 500 Patientenbetten (Ohne Unikliniken/Maximalversorger) - Kliniken mit 250 bis 499 Patientenbetten (Ohne Unikliniken/Maximalversorger) - Kliniken mit < als 250 Patientenbetten (Ohne Unikliniken/Maximalversorger |
| **C** | **Personalstruktur Krankenhausbegehungen** | |
| C | Bezogen auf Regelbegehungen: Haben Sie ein organisatorisch getrenntes Team zuständig nur für Krankenhäuser oder ein gemeinsames Team zuständig für Krankenhäuser und weitere Gesundheitseinrichtungen (z.B. Pflegeeinrichtungen)? | *Einfachauswahl und Kommentarfeld*   - Getrennte Teams - Gemeinsames Team - Sonstiges (Kommentarfeld) |
| C | Wie viele Personen sind an Ihrem Gesundheitsamt durchschnittlich im Jahr für Regelbegehungen von Krankenhäusern zuständig (unabhängig von der Berufsgruppe)? Bitte schätzen Sie in Vollzeitkräften. | *Einfachauswahl und Kommentarfeld*  <2  2 - <3  3 - <4  4 - <5  5 - <66  6 - <7  7 - <8  8 - <9  ≥ 9  Alternativ: Beschreiben Sie hier (Kommentarfeld) |
| C | Welche Berufsgruppen sind von Seiten des Gesundheitsamtes an der Regelbegehung von Krankenhäusern beteiligt? | *Mehrfachoption mit Kommentarfeld*   - Hygienefachkraft - Hygiene-/Gesundheitsaufseher - Auszubildende, bitte Fachrichtung(en) angeben - Hygieneingenieur - Arzt - Exam. Gesundheits- und Krankenpfleger (Hygienefachkräfte ausgenommen) - Sonstiges, bitte angeben |
| C | Bezogen auf ärztliches Personal: Wie hoch schätzen Sie den Zeitaufwand in Ihrer Behörde, der durchschnittlich für Regelbegehungen von Krankenhäusern aufgewendet wird? Hierbei ist der Gesamtaufwand einschließlich Vor- und Nachbereitung in Vollzeitarbeitstagen pro Jahr anzugeben. *Zwei Beispielrechnungen mit der Annahme, dass ein Jahr abzüglich Wochenenden, Feiertage und 30 Urlaubstage ca. 220 Vollzeitarbeitstage hat.*  *1. Beispielrechnung: 1 Vollzeitkraft ist in 50% Ihrer Tätigkeit mit Krankenhausbegehungen befasst 220 x 0,5 = 110 Tage 2 Vollzeitkräfte sind ausschließlich mit Krankenhausbegehungen befasst 220 x 2 = 440 Tage*  *2. Beispielrechnung: Eine Krankenhausbegehung benötigt bei Ihnen im Schnitt 5 Vollzeit-Arbeitstage. In Ihrem Kreis befinden sich 10 Kliniken, die von Ihnen begangen werden. 5 x 10 = 50 Tage* | *Einfachauswahl und Kommentarfeld*  ≤ 5 Tage  6 – 10 Tage  11 – 20 Tage  21 - 30 Tage  31 – 50 Tage  51 – 70 Tage  71 – 100 Tage  101 – 150 Tage  151 – 220 Tage > 220 Tage/Jahr - Alternativ: Beschreiben Sie hier (Kommentarfeld) |
| C | Bezogen auf nicht-ärztliches Personal: Wie hoch schätzen Sie den Zeitaufwand in Ihrer Behörde, der durchschnittlich für Regelbegehungen von Krankenhäusern aufgewendet wird? Hierbei ist der Gesamtaufwand einschließlich Vor- und Nachbereitung in Vollzeitarbeitstagen pro Jahr anzugeben. *Zwei Beispielrechnungen mit der Annahme, dass ein Jahr abzüglich Wochenenden, Feiertage und 30 Urlaubstage ca. 220 Vollzeitarbeitstage hat.*  *1. Beispielrechnung: 1 Vollzeitkraft ist in 50% Ihrer Tätigkeit mit Krankenhausbegehungen befasst 220 x 0,5 = 110 Tage 2 Vollzeitkräfte sind ausschließlich mit Krankenhausbegehungen befasst 220 x 2 = 440 Tage*  *2. Beispielrechnung: Eine Krankenhausbegehung benötigt bei Ihnen im Schnitt 5 Vollzeit-Arbeitstage. In Ihrem Kreis befinden sich 10 Kliniken, die von Ihnen begangen werden. 5 x 10 = 50 Tage* | *Einfachauswahl und Kommentarfeld*  ≤ 5 Tage  6 – 10 Tage 11 – 20 Tage 21 - 30 Tage 31 – 50 Tage 51 – 70 Tage 71 – 100 Tage 101 – 150 Tage 151 – 220 Tage > 220 Tage/Jahr Alternativ: Beschreiben Sie hier (Kommentarfeld) |
| **D** | **Ablauf und organisatorische Aspekte der Regelbegehung von Krankenhäusern** | |
| D | In welchem Rhythmus wurde bei Ihnen bisher die Regelbegehung eines Universitätsklinikums/Maximalversorgers durchgeführt? Hiervon ist die Corona-Pandemie-Phase ausgenommen. *Bitte den tatsächlichen Rhythmus und nicht die vorgegebene Frequenz angeben* | *Einfachauswahl*   - Keine Universitätsklinik/Maximalversorger im Einzugsgebiet - Alle 3 Jahre oder seltener - Alle 2 Jahre - 1x im Jahr - 2x im Jahr - - Häufiger als 2x im Jahr |
| D | In welchem Rhythmus wurde bei Ihnen bisher die Regelbegehung von Krankenhäusern (ohne Universitätskliniken/Maximalversorger) durchgeführt? Hiervon ist die Corona-Pandemie-Phase ausgenommen. *Bitte den tatsächlichen Rhythmus und nicht die vorgegebene Frequenz angeben* | *Einfachauswahl*   - Alle 3 Jahre oder seltener - Alle 2 Jahre - 1x im Jahr - 2x im Jahr - - Häufiger als 2x im Jahr |
| D | Wie strukturieren Sie die Regelbegehungen von Universitätskliniken/Maximalversorger? | *Mehrfachauswahl mit Kommentarfeld*   - Jede/s Klinik/Institut des Universitätsklinikums/Maximalversorgers wird einzeln begangen - Das Universitätsklinikum/der Maximalversorger wird als Ganzes begangen - Zentren, die mehrere Kliniken umfassen (z.B. Kopfzentrum, Eltern-Kind-Zentrum u.a.) werden zusammen begangen - Sonstiges (Kommentarfeld) |
| D | Welche der folgenden Aussagen zur thematischen Gestaltung der Regelbegehungen von Krankenhäusern (Ohne Unikliniken/Maximalversorger) treffen für Sie zu? Bei der Regelbegehung werden gezielt … | *Mehrfachauswahl mit Kommentarfeld*   - ...Problembereiche aus der letzten Regelbegehung überprüft. - ...neue und/oder sanierte Bereiche überprüft. - ...wiederkehrend die gleichen, allgemein relevanten Inhalte überprüft. - ...für sämtliche Krankenhäuser übergreifend ein Schwerpunktthema festgelegt, das überprüft wird (z.B. Jahr 2020-Reinigung, 2021-Notaufnahmen, …) - … Sonstige Begehungskonzepte angewandt: (Kommentarfeld) |
| D | Welche der folgenden Aussagen zur thematischen Gestaltung der Regelbegehungen von Unikliniken/Maximalversorger treffen für Sie zu? Bei der Regelbegehung werden gezielt… | *Mehrfachauswahl mit Kommentarfeld*   - ...Problembereiche aus der letzten Regelbegehung überprüft. - ...neue und/oder sanierte Bereiche überprüft. - ...wiederkehrend die gleichen, allgemein relevanten Inhalte überprüft. - ...für sämtliche Krankenhäuser übergreifend ein Schwerpunktthema festgelegt, das überprüft wird (z.B. Jahr 2020-Reinigung, 2021-Notaufnahmen, …) - … Sonstige Begehungskonzepte angewandt: (Kommentarfeld) |
| D | Informieren Sie das Krankenhaus über den exakten Termin der Regelbegehung? | *Einfachauswahl*   - Ja, es wird der Tag der Begehung mitgeteilt (z.B. 05. Oktober 2024) - Ja, es wird die Woche, in der die Begehung stattfindet, mitgeteilt (z.B. KW 23) - Ja, es wird ein grober Zeitraum, in dem die Begehung stattfindet, mitgeteilt (z.B. März) - Nein, wir teilen vorab nicht mit, wann eine Begehung stattfinden wird |
| D | Wie weit im Voraus (schätzungsweise) informieren Sie das Krankenhaus über den Begehungstermin | *Einfachauswahl*   - 1 Woche im Voraus - 2 Wochen im Voraus - 3 bis 4 Wochen im Voraus - 1 bis 2 Monate im Voraus - Mehr als 3 Monate im Voraus |
| D | Informieren Sie das Krankenhaus vorab über den Inhalt der Regelbegehung? | *Einfachauswahl*   - Ja - Nein |
| D | Werden vor der Regelbegehung Unterlagen angefordert? | *Einfachauswahl mit Kommentarfeld*   - Nein - Ja, welche (Kommentarfeld) |
| D | In welcher Form lassen Sie sich die Unterlagen zukommen? | *Einfachauswahl mit Kommentarfeld*   - E-Mail - Geschützte Cloud - Verschlüsselte ZIP-Dateien - Fax - Ausdrucke per Post - Sonstiges (Kommentarfeld) |
| D | Erfolgt eine separate Vorbesprechung mit dem Krankenhaus vor der Regelbegehung? | *Einfachauswahl*   - Ja - Nein |
| D | Welche Themen werden bei dieser Vorbesprechung normalerweise besprochen? | *Mehrfachauswahl*   - Fehlende Unterlagen - Bezug zur letzten Begehung - Surveillancedaten - Auffällige Ergebnisse von Umgebungsuntersuchungen - Ausbrüche - Qualifikation und /oder Fortbildungen des Hygienepersonals - Nachweise zu Hygiene-Fortbildungen des Klinikpersonals - Zertifikate (z.B. Wäscherei RAL-Zertifizierung etc.) - Sonstiges: (Kommentarfeld) |
| D | Gibt es bei Ihnen einheitliche Vorgaben, welche Personen seitens des Krankenhauses regulär bei der Regelbegehung anwesend sein sollen (gemäß Ihren Vorgaben)? | *Einfachantwort*   - Ja - Nein |
| D | Welche Personen des Krankenhauses sollen regulär bei der Regelbegehung anwesend sein (gemäß Ihren Vorgaben)? | *Matrix nach der Auswahl:*   - Ja, Person soll anwesend sein - Ja, Person soll erreichbar sein bei Bedarf - Nein   *Antworten:*   - Krankenhaushygieniker - Hygienefachkraft - Hygienebeauftragte Ärzte - Ärztlicher Direktor - Kaufmännische Geschäftsführung - Pflegedienstleitung - Mitarbeitende aus der Technik - Arbeitssicherheit - Betriebsmedizin - Reinigungsdienst |
| D | Welche weiteren Personen des Krankenhauses außer die zuvor Genannten sollen regulär bei der Regelbegehung anwesend sein (gemäß Ihren Vorgaben)? | *Freitext* |
| D | Welche Personen des Krankenhauses sollten Ihrer Meinung nach regulär bei der Regelbegehung anwesend sein? | *Matrix nach der Auswahl:*   - Ja, Person soll anwesend sein - Ja, Person soll erreichbar sein bei Bedarf - Nein   *Antworten:*   - Krankenhaushygieniker - Hygienefachkraft - Hygienebeauftragte Ärzte - Ärztlicher Direktor - Kaufmännische Geschäftsführung - Pflegedienstleitung - Mitarbeitende aus der Technik - Arbeitssicherheit - Betriebsmedizin   Reinigungsdienst |
| D | Welche weiteren Personen des Krankenhauses außer die zuvor Genannten sollten Ihrer Meinung nach regulär bei der Regelbegehung anwesend sein? | *Freitext* |
| D | Nutzen Sie während der Begehung standardisierte Checklisten oder Protokolle? | *Einfachauswahl*   - Ja - Nein |
| D | Wenn ja: Von wem wurden diese hauptsächlich erstellt? | *Einfachauswahl mit Kommentarfeld*   - Eigenes Gesundheitsamt - Anderes Gesundheitsamt - Eigene Landesgesundheitsbehörde - Andere Behörde (Kommentarfeld) - Sonstiges (Kommentarfeld) |
| D | Wie dokumentieren Sie während der Begehung im Krankenhaus? | *Mehrfachauswahl mit Kommentarfeld*   - Gar nicht während der Begehung - Papier - Elektronisch via Tablet oder Handy - Elektronisch via Laptop/Notebook - Fotodokumentation - Diktat/Audioaufnahme - Sonstiges (Kommentarfeld) |
| D | Wenn elektronisch: Welche Software nutzen Sie für die elektronische Dokumentation? | *Mehrfachauswahl mit Kommentar*   - Office-Anwendung (Word, Excel, ...) - PDF-Formular - Eigene Softwarelösung (Bitte im Kommentarfeld angeben) - Sonstiges (Bitte im Kommentarfeld angeben) |
| D | Nutzen Sie eine Fotodokumentation? | *Einfachauswahl*   - Ja, regulär - Ja, nur in Ausnahmefällen - Nein |
| D | Wie werden die Ergebnisse der Begehung kommuniziert? | *Mehrfachauswahl mit Kommentarfeld*   - Direkt im Nachgang mündlich vor Ort - In einer separaten Nachbesprechung an einem anderen Tag (telefonisch, vor Ort, sonstige Form) - Schriftlich im Bericht - Sonstiges (Kommentarfeld) |
| D | Erstellen Sie nach der Begehung einen Bericht oder Vergleichbares? | *Einfachauswahl*   - Ja - Nein |
| D | Wird dieser Bericht an das Krankenhaus gegeben? | *Einfachauswahl*   - Ja, der komplette Bericht wird an das Krankenhaus gegeben - Ja, aber nur ein Teil des Berichts mit den notwendigen Maßnahmen wird an das Krankenhaus gegeben - Nein |
| D | Werten Sie die Ergebnisse der Regelbegehungen systematisch aus? | *Einfachauswahl*   - Ja, intern zur eigenen Einschätzung innerhalb der Behörde - Ja, mit anonymen Feedback an die Einrichtung - Nein |
| D | Bezogen auf die vorherige Frage: Wie vergleichen Sie die Krankenhäuser untereinander? | *Freitext* |
| **E** | **Inhalt von Regelbegehungen** | |
| E | Wie wichtig erachten Sie bei der Regelbegehung von Krankenhäusern die Abfrage bzw. Kontrolle folgender Punkte: | *Likert-Skala nach der Auswahl:*   - Sehr wichtig - Eher wichtig - Indifferent - Eher unwichtig - Sehr unwichtig   *Antworten*   - Personelle Struktur, z.B. Änderungen in Leitungspositionen - Strukturdaten, z.B. Bettenzahl - Organisatorische Umstrukturierungen - Geplante u/od. durchgeführte Baumaßnahmen - Umgesetzte Maßnahmen aus den Vorjahren - Sitzungsprotokolle der Hygienekommissionssitzungen - Hygieneplan gesamt - Hygieneplan in Auszügen - Wasser-Hygieneplan / Water Safety Plan - Notversorgungspläne (z.B. Strom, Wasser) - Hitzeschutzplan - Qualifikationsnachweise des Hygienepersonals - Weisungsbefugnis des Hygienepersonals vorhanden - Geplanter und/oder reeller Stellenbesetzungsschlüssel des Hygienepersonals - Stellenschlüssel des Krankenhauspersonals allgemein (Pflege, Ärzte) - Hygiene-Schulungsnachweise des Krankenhauspersonals - Stichprobenartige Überprüfung, ob dem Personal der Hygieneplan bekannt ist - Surveillance gemäß § 23: Erreger- und Resistenzstatistik - Surveillance gemäß § 23: Nosokomiale Infektionen (KISS, KISS analog oder sonstige) - Erfassung & Bewertung Händedesinfektionsmittelverbrauch - Erfassung & Bewertung Antibiotikaverbrauch - Festlegung der Screeningmaßnahmen - Adäquate Isoliermaßnahmen beim Umgang mit multiresistenten Erregern Adäquate Persönliche Schutzausrüstung beim Umgang mi - Adäquate Persönliche Schutzausrüstung beim Umgang mit multiresistenten Erregern - Personelle ABS-Struktur - Reinigung intern oder durch externe Firma - Personelle Aufstellung des Reinigungspersonals - Zustand und Lagerung von Wischbezügen - Inhaltliche Korrektheit von Reinigungs- und Desinfektionsplänen - Wäscherei: Intern oder externe Firma - RAL-Zertifizierung der Wäscherei - Verfahrensanweisung zu Umgang mit verstorbenen infektiösen Patienten - Turnus und Ergebnisse qualitätssichernder Routineuntersuchungen (Umgebungsuntersuchungen - Turnus und Ergebnisse von Trinkwasseruntersuchungen - Wartung und Prüfung von Raumlufttechnischen Anlagen - Lagerungsbedingungen für Sterilgut und Medizinprodukte |
| E | Welche besonders wichtigen, noch nicht genannten Inhalte prüfen Sie bei der Regelbegehung? (Allgemein oder in besonderen Bereichen, z.B. Intensivstation) | *Freitext* |
| E | Welche Abteilungen/Bereiche erachten Sie, bezogen auf Ihre Krankenhäuser, als besonders wichtig bei der Regelbegehung? | *Likert-Skala nach der Auswahl:*   - Sehr wichtig - Eher wichtig - Indifferent - Eher unwichtig - Sehr unwichtig - Nicht vorhanden   *Antworten*   - Innere Medizin - Chirurgie - Anästhesie - Geburtshilfe/Gynäkologie - Orthopädie/Unfallchirurgie - Urologie - HNO - Dermatologie - Augenheilkunde - Mund-Kiefer-Gesichtschirurgie - Geriatrie - Neurologie - Psychiatrie/Psychosomatik - Neuro-Chirurgie - Thorax-/Kardiochirurgie - Kinderchirurgie - Pädiatrie - Neonatologie - Infektiologie/Isolierstation - Sonderisolierstation/Hochisolierstation - Hämato-Onkologie/KMT - Transplantationszentrum - Plastische Chirurgie - Verbrennungszentrum - Strahlentherapie/Nuklearmedizin - Intensivstation/IMC - Notaufnahme - Operationseinheit - Endoskopie - Herzkatheter - Radiologie - Blutbank/Transfusionsmedizin - Reinigung - Küche - Lager und Logistik - Sterilisation/Zentrale Aufbereitungseinheiten - Dialyse - Milchküche - Apotheke (intern) - Zahnklinik |
| E | Welche Abteilungen/Bereiche, die nicht in der vorherigen Frage genannt wurden, erachten Sie als besonders wichtig bei der Regelbegehung von Krankenhäusern in Ihrem Kreis/Ihrer Stadt? | *Freitext* |
| E | Zur Aufbereitung von Medizinprodukten: Prüfen Sie gezielt die Aufbereitungsprozesse und Validierung im Rahmen der Regelbegehung? | *Einfachauswahl mit Kommentarfeld*   - Ja - Nein, im Rahmen anderer Untersuchungen durch unser Gesundheitsamt - Nein, da hierfür eine andere Behörde zuständig ist, nämlich (Kommentarfeld) - Nein, da anderer Grund (Kommentarfeld) |
| E | Begehen Sie klinikinterne Apotheken? | *Einfachauswahl mit Kommentarfeld*   - Ja - Nein, da nicht vorhanden - Nein, da hierfür eine andere Behörde zuständig ist (Kommentarfeld) - Nein, da anderer Grund (Kommentarfeld) |
| **F** | **Zusatz - MRE-Netzwerke** | |
| F | Sind Sie als Gesundheitsamt Mitglied eines MRE-Netzwerks? | *Einfachauswahl mit Kommentarfeld*   - Ja, derzeit aktives MRE-Netzwerk (Name des Netzwerks: Kommentarfeld) - Nein, derzeit inaktives MRE-Netzwerk (Name des Netzwerks: Kommentarfeld) - Nein - Weiß ich nicht |
| F | Wenn ja bzw. inaktiv: Wie viele Städte/Kreise sind innerhalb Ihres MRE-Netzwerkes zusammengeschlossen? | *Einfachauswahl*   - 1 - 2 - 3-5 - 6-9 - Mehr als 9 |
| **G** | **Feedback** | |
| G | Möchten Sie uns etwas mitteilen, das Sie für wichtig erachten und nicht im Fragebogen thematisiert wurde? | *Freitext* |
